# The Psychosocial Effects of Lockdown Due to the COVID-19 Pandemic on Children in 2021

**DOI:** 10.7759/cureus.53614

**Published:** 2024-02-05

**Authors:** Puneet Anand, Rutuja S Patil, Piyush Puri, Sanjivani Patil

**Affiliations:** 1 Pediatrics, Icahn School of Medicine at Mount Sinai, Elmhurst Hospital Center, New York City, USA; 2 Medicine, Bharati Vidyapeeth Medical College Pune, Pune, IND; 3 Internal Medicine, Adesh Institute of Medical Science and Research, Bathinda, IND; 4 Community Medicine, Bharati Vidyapeeth Medical College, Pune, IND

**Keywords:** covid-19 pandemic, pandemic, public psychiatry, child and adolescent psychiatry, psychosocial stress, covid-19

## Abstract

Objectives

The COVID-19 pandemic led to a nationwide lockdown that isolated numerous children and adolescents, significantly affecting their mental well-being. Therefore, this study aimed to assess the challenges faced by children during the pandemic and identify the potential contributing factors. Additionally, given the existing concerns surrounding the mental health of female children and adolescents, our study aimed to investigate the presence of sex-based disparities in children's observed emotional and behavioral difficulties during the COVID-19 lockdown.

Methods

Participants in this study were parents of children aged 6-17 years. An observational cross-sectional study was conducted through verbal administration of a validated semi-structured questionnaire, the Parent Report Measure, from June 2021 to August 2021 at a nearby community health center. The questionnaire collected socio-demographic details and utilized the Strengths and Difficulties Questionnaire (SDQ), a screening tool to assess children's emotional and behavioral aspects during the six-month lockdown period. The responses were then analyzed using appropriate statistical tools like SPSS statistics, and cumulative SDQ scores were used to categorize participants.

Results

Out of the total 280 responses analyzed, the prosocial subscale exhibited the highest number of abnormal responses, 73 (26.1%), followed by conduct (42; 15%), hyperactivity (41; 14.6%), and peer (41; 14.6%) subscales. These results indicated that children displayed decreased empathy towards others, restlessness, fidgetiness, reduced attention span, frequent tantrums, and a preference for solitary activities. Furthermore, there was a significant association between abnormal subscale scores and the sex of children. Females showed a considerably higher prevalence of emotional problems (172; 61.5%) than males. Among the behavioral responses, a more significant proportion of females displayed abnormal scores in the conduct subscale (170; 60.7%), while abnormal hyperactivity scores were more frequently observed among males (178; 63.4%). Regarding the peer problems subscale, the proportion of females was slightly higher than males (150; 53.7%) and nearly equal in the prosocial domain. A more significant proportion of females displayed abnormal scores for overall difficulties 144 (51.5%), indicating a notable sex-based disparity.

Conclusion

Our findings highlight the pandemic's significant impact on many children's psychological well-being. The results emphasize the need for targeted interventions to address the mental health concerns that arose in this population. The observed disparity in emotional and behavioral difficulties among female children is particularly concerning, highlighting the need for gender-sensitive support and care. Various strategies can be helpful, such as virtual support groups, indoor recreational activities, reduced screen time and excessive news consumption, and effective communication with parents. Furthermore, our study also indicates the need to dive deep into such areas of pediatric research to understand and plan timely interventions.

## Introduction

As COVID-19 rapidly spread across the globe, governments implemented full lockdown measures, resulting in serious consequences on the economy, health, and psychosocial well-being of societies. Among the affected population, children exhibited heightened vulnerability due to factors such as their age, socioeconomic status, and mental development [1].

While the negative consequences were evident across all age groups, children, in particular, experienced some unique challenges. With the schools being shut and restrictions placed on outdoor activities, many researchers observed a surge in stressors impacting the health of both children and adults [2]. Adolescents, in particular, exhibited a higher propensity to seek solace and information through social media platforms, indicating a shift in coping mechanisms during the pandemic [3]. This was reflected in the increased volume of calls from helplines such as CHILDLINE, which experienced a fifty percent spike during the lockdown period [4].

Children respond to fear and anxiety differently than adults [5]. Some may turn to television for distraction, while others may withdraw and isolate themselves. These reactions can often be challenging for patients to understand, leading to misinterpretation and further exacerbation of frustration and anxiety.

Hence, this study was undertaken to assess the psychosocial effects of lockdown among school-aged children and adolescents. Specifically, the study aims to utilize the Strengths and Difficulties Questionnaire (SDQ) to assess the psychosocial behavior of children and adolescents during the lockdown period.

## Materials and methods

This observational, cross-sectional study was conducted over two months at the selected urban health center affiliated with Bharati Vidyapeeth Medical College, Pune, India. The study consisted of 272 participants, determined through the following sample size calculation formula, incorporating a prevalence rate (p) of 26.9% and a desired effect size (d) set at 20% of the prevalence rate: n= Z^2^ x p x (1-p) / d^2^, where Z is Z-score.

The selection criteria included parents of children aged 6-17 years who were willing to give consent, excluding children with severe learning disabilities and parents unable to understand Hindi or English.

Data was collected using a validated semi-structured parent-report measure questionnaire, including sociodemographic details and a Strength and Difficulty Questionnaire (SDQ) [6]. Data collection occurred through structured interviews with parents or guardians, obtaining informed consent.

SDQ is a well-established brief emotional and behavioral screening tool for children and young people, consisting of 33 questions. The initial 25 questions are divided into five subscales: emotional symptoms, conduct problems, hyperactivity/ inattention, peer relationship problems, and prosocial behavior. Individual responses were coded on a scale of 0 (not true) to 2 (certainly true), which allowed for the categorization of participants based on their cumulative SDQ scores into abnormal (17-30), borderline (14-16), and normal (below 13).

Plan of statistical analysis of results

The data was analyzed using SPSS version 20 (IBM Inc., Armonk, New York); for qualitative data, various rates, ratios, and percentages (%) were calculated. For quantitative data, the mean SD, mean, etc., were calculated. For qualitative data, wherever applicable, tests like the chi-square test and for quantitative data, tests like t-test/ANOVA were used to compare variables. A p-value of 0.05 was considered significant.

## Results

This study involved the participation of 280 parents who completed the questionnaire and the consent form. Complete data were analyzed for the children of these 280 participants, representing a 100% response rate. The demographics can be seen from (Table 1).

**Table 1:** Descriptive analysis of the socioeconomic data

Parameter	Number of participants	Maximum	Minimum	Mean	SD
Age of child in years	280	3	38	11.20	4.27
Number of children	280	7000	5	2.41	1.04
Family income	280	1	3000000	106485.71	301000.03
Screen time	280	0	14	6.75	2.85
Play time: outdoor/indoor	280	0	8	2.80	2.16
Emotional state	280	0	10	1.57	2.18
Conduct state	280	0	9	1.49	2.00
Hyperactivity state	280	0	10	2.87	2.95
Peer problem	280	0	9	1.50	1.86
Prosocial	280	0	10	6.65	3.34
Total difficulties score	280	0	28	7.44	6.88
Parent report	280	0	10	0.80	1.95

The study revealed that the average annual family income was 106485.71, with an SD of 301000.03. Moreover, 99.6% (278) of the mothers had received at least primary education, while 14.3% (40) of the population had completed their post-graduation studies. All fathers had received primary education, with 15% of the participants having completed their post-graduation studies. The average number of children per parent was found to be 2.41, with an SD of 1.04.

The study population primarily consisted of females 163 (58.2%), while males constituted 117 (41.8%) of the sample. The participants had a mean age of 11.20 years with an SD of 4.27. Furthermore, the study also identified that 20 (7.2%) of the children had significant developmental delays.

Amidst the lockdown, a significant majority of the participants, specifically 257 (91.8%), reported having access to some form of education facility. On average, the participants reported spending approximately 6.75 hours, with an SD of 2.85, engaged in screen-based activities, encompassing both educational and recreational purposes. The maximum playtime, considering both outdoor and indoor activities, was found to be eight hours, with an average of 2.80 hours and an SD of 2.16.

The categorization of the Strengths and Difficulties Questionnaire scores of children and the corresponding findings are presented in (Table 2).

**Table 2:** SDQ scoring results SDQ - Strengths and Difficulties Questionnaire

SDQ scoring parameter (completed by parents)	Abnormal (%)	Borderline (%)	Normal (%)
Hyperactivity/inattention	14.6	7.9	77.5
Emotional problem	10	7.1	82.9
Conduct problem	15	8.9	76.1
Peer problems	14.6	8.9	76.4
Prosocial problem	26.1	6.8	67.1
Total difficulties	7.1	5.4	87.5

Our analysis revealed that the highest percentage of abnormal scores was observed in the prosocial problems subscale (73; 26.1%). This indicates that a considerable number of children encountered difficulties or displayed limited behavioral tendencies towards considering other people's feelings, sharing toys/food with other children, being kind to younger individuals, volunteering to help others, and providing help when someone is hurt. The lack of in-person interactions with peers of the same age may have contributed to the limited exposure and experience that is necessary to develop helpful and empathetic attitudes in these situations.

The second highest percentage of abnormal scores, 42 (15%), was identified in the conduct problems subscale. Parents reported that their children demonstrated disobedience and frequent tantrums, as well as behavioral tendencies such as lying and stealing. These observations suggest a potential influence of inadequate moral and values education available at the school level, as well as limited interaction and communication with parents, teachers, and other influential role models.

Additionally, a p-value of <0.01 was accepted as statistically significant for abnormal scores in the hyperactivity problems subscale observed in 41 (14.6%) of the participants. Parents reported an increased prevalence of restlessness, fidgeting, difficulties maintaining overall concentration, and a propensity to get distracted easily among their children. These findings can be attributed to the reduced playtime and a relatively inactive lifestyle experienced by children during the lockdown, resulting in limited avenues to channel their surplus energy. Furthermore, the increased use of social media and screen time likely contributed to a decline in concentration levels.

Significant abnormal scores were also noted in the peer problems subscale (41; 14.6%). Parents reported that their children had fewer friends and tended to engage in solitary activities. These findings suggest limited opportunities for social interactions and potential challenges in establishing friendships during the lockdown.

A p-value of <0.043 was accepted as statistically significant for abnormal scores were also observed in the emotional problem subscale in a significant proportion of children 28 (10%). These scores indicated that children experienced feelings of unhappiness, worry, anxiety, and distress. The uncertainty related to the pandemic, including the effects of the lockdown and changes in routine, along with other factors such as scarcity of resources or the presence of an ill parent or relative, may have contributed to these emotional challenges experienced by the children during the lockdown.

We also conducted a chi-square test to analyze the association between abnormal subscale scores and the sex of the participants. The results revealed that there were significant variations in the prevalence of emotional problems between the sexes. Specifically, a higher proportion of female children exhibited emotional problems.

Among the children with abnormal scores for conduct, the majority, 170 (60.7%), were females. On the other hand, abnormal scores for hyperactivity were more prevalent in males (177; 63.4%).

In terms of peer problems, a slightly higher proportion of females, 150 (53.7%), displayed abnormal scores compared to males, 130 (46.3%). The subscale of prosocial behavior showed relatively equal abnormal scores for both females (142; 50.7%) and males (138; 49.3%). Furthermore, a significant proportion of females, 172 (61.5%), demonstrated abnormal scores on the emotional subscale, indicating a higher prevalence of emotional problems in this group. Additionally, when considering the overall difficulties score, a higher proportion of abnormal scoring was observed amongst females (144; 51.5%) compared to males (136; 48.5%).

These findings suggest a sex-based disparity among the study participants, with females showing a higher proportion of abnormal scores in the conduct, peer problems, and the emotional subscale. Further research is needed to gain a deeper understanding of the underlying factors contributing to this observed difference and to develop targeted interventions aimed at addressing the psychosocial well-being of children.

## Discussion

During the severe acute respiratory syndrome (SARS) epidemic, research conducted by Brooks et al. revealed that children who were quarantined reported four times higher stress scores for post-traumatic stress disorder (PTSD) compared to those who were not quarantined [7]. A study conducted in India during the COVID-19 pandemic yielded comparable results [8]. Additionally, studies conducted in China found that young individuals reported increased depressive and anxiety symptoms [9], along with negative psychological consequences, as a result of the COVID-19 pandemic [10]. Various stressors such as school closures, fear of infection, strained interpersonal relationships within families, mental health and anxiety levels of caregivers, academic pressure, education being compromised, and financial insecurity were identified as factors potentially affecting children's well-being [11].

The lockdown was implemented to ensure the protection of susceptible individuals and to mitigate the spread of the virus [12], but it inadvertently has had negative implications for the mental health and psychological well-being of children [13]. Our study aimed to analyze the impact of lockdown, assess its magnitude, and identify contributing factors to the psychological well-being of children.

The reduction in outdoor activities emerged as a prominent factor contributing to psychological distress in children [14]. The nationwide lockdown and closure of schools limited children's access to outdoor playtime and social interactions with peers of the same age [15]. Additionally, symptoms associated with severe acute respiratory distress increased anxiety and worry, suggesting that infectious conditions can also influence the psychological well-being of children, similar to other traumatic experiences [6].

In line with our findings, previous research conducted in North East India also reported that female children experienced more psychological distress [16]. This gender disparity could be attributed to ingrained patriarchal societal norms that give less attention, care, and opportunities to female children, illiteracy of the parents, taxing domestic work in low socioeconomic households, neglect towards hygienic living conditions, and exploitation of their basic human rights [17].

The scarcity and uncertainty surrounding basic resources such as food and water [18], as well as parental job loss, showed that economic and financial stressors significantly add to the psychological burden of children [19] and aggravate many symptoms such as frustration, anxiety, anger, and irritability [20].

The lack of support and communication from parents and other family members most likely leads to these adverse symptoms, indicating that more attention is required towards the importance of psychological support in children [21]. Additionally, the fear generated by the news and social media [22], the inundation of negative information [22], parental stress [23], and the lack of indoor hobbies [24] may also have directly or indirectly impacted children's mental well-being.

Some advantages of our study include its comprehensive approach to analyzing the effects of the lockdown on children's mental and psychological well-being, as well as being the first study, to the best of our knowledge, to provide data on sex differences in children in this context.

Additionally, by using validated questionnaires and conducting in-person interviews, we obtained detailed data that provided a holistic understanding of the participants' experiences. The 100% response rate reflects a high level of participant engagement, ensuring a robust dataset for analysis. Conducting the study during the peak of the COVID-19 pandemic allowed us to capture real-time experiences and minimize recall bias.

However, there are several limitations to our study. As an observational cross-sectional study, our findings provide a snapshot of the impact of lockdown on children's mental well-being but do not capture its long-term effects. The use of parent-completed questionnaires as the primary assessment tool may introduce subjectivity and response bias.

Another limitation is the potential for selection bias, as our study was conducted in a specific location or region, which could limit the generalizability of our findings to other populations with different socio-cultural contexts. Future research should aim to include a more diverse and representative sample to enhance the external validity of the findings.

Despite these limitations, our study provides valuable insights into the psychological impact on children's well-being, particularly in terms of sex differences. The inclusion of sex as a factor of analysis expands the existing knowledge base and highlights the differential effects of lockdown on the psychological health of male and female children. The in-person interviews allowed for a more nuanced understanding of their experiences, while the inclusion of socioeconomic factors strengthened the relevance and applicability of our findings to inform targeted interventions and support strategies for children and families affected by similar circumstances. 

The nationwide lockdown had a significant negative impact on the mental health of children and adolescents in India, and young female children were affected to a considerable extent. Even before the pandemic, mental health issues among young girls were a cause of concern, and the current situation has emphasized the need for intervention [25].

Limitations

The study was conducted in a specific location or region, which may limit its generalizability to other populations with different socio-cultural contexts. Future research should aim to include a more diverse and representative sample to enhance the external validity of the findings.

The use of parent-completed questionnaires as the primary assessment tool may introduce subjectivity and response bias. It's important to acknowledge that parents' perceptions of their children's behavior may vary, and self-report measures from children themselves could provide a more comprehensive understanding of their experiences.

As an observational cross-sectional study, the findings provide a snapshot of the impact of lockdown on children's mental well-being but do not capture its long-term effects. Longitudinal studies would be beneficial to track changes in children's mental health over time and identify potential causal relationships.

## Conclusions

Our study identified various aspects of children's mental well-being that were affected by the lockdown. Furthermore, our study highlights the need for increased research focus on the mental health of pediatric age groups. While psychological distress in adults has received substantial attention, there are relatively fewer studies and interventions specifically tailored to children's well-being. Therefore, prioritizing research in this area will enable the development of timely and effective interventions to address the unique mental health challenges faced by children and adolescents.
